# The effect of vildagliptin versus metformin on hepatic steatosis in type 2 diabetic patients: a randomized controlled trial

**DOI:** 10.1186/s40360-024-00818-7

**Published:** 2024-12-13

**Authors:** Asmaa S. Mohamed, Hosam M. Ahmad, Mohammed A. Sharawy, Fatma M. M. Kamel

**Affiliations:** 1 Clinical Pharmacy and Pharmacy Practice Department, Faculty of Pharmacy, Port said University, Port said, Egypt; 2 Internal Medicine and Biomedical Chemistry Departments, Egypt Ministry of Health and Population, Minia, Egypt; 3 Internal Medicine Department, Faculty of Medicine, Minia University, Minia, Egypt

**Keywords:** Vildagliptin, Metformin, Hepatic steatosis, Type 2 diabetes mellitus

## Abstract

**Background:**

The risk of hepatic steatosis (HS) is elevated in patients with type 2 diabetes mellitus (T2D). Antidiabetic medications may contribute to the prevention or treatment of HS. This study aimed to compare the effects of vildagliptin and metformin on hepatic steatosis in newly diagnosed T2D patients, using the Hepatic Steatosis Index (HSI) and ultrasound grading.

**Methods:**

The study included 246 newly diagnosed T2D patients who were randomly assigned to two groups. The first group (117 patients) received 50 mg of vildagliptin orally twice daily. The second group (129 patients) received 500 mg of metformin orally twice daily with meals, and the dosage could be gradually increased by 500 mg per week, up to a maximum daily dose of 2000 mg. Baseline and 6-month follow-up assessments included fasting blood glucose (FBG), HbA1c, weight, body mass index (BMI), waist circumference (WC), hip circumference (HC), the Hepatic Steatosis Index (HSI), and hepatic steatosis grading via ultrasound.

**Results:**

Both groups showed significant improvements in FBG, HbA1c, weight, BMI, WC, HC, HSI, and ultrasound grading of hepatic steatosis from baseline to the 6-month follow-up (*p* < 0.001). The metformin group demonstrated significantly greater reductions in weight and BMI compared to the vildagliptin group (*p* = 0.001 and *p* = 0.009, respectively). However, there was no significant difference between the two groups in terms of hepatic steatosis improvement on ultrasound. Correlation analysis revealed that HSI was significantly associated with HbA1c, BMI, WC, and HC (*p* < 0.001 for all), as well as FBG (*p* = 0.008), but not with age. The lipid profile, particularly total cholesterol and LDL, was identified as a stronger predictor of hepatic steatosis, based on high AUC, sensitivity, and specificity values.

**Conclusion:**

Both vildagliptin and metformin are effective in improving glycemic control in newly diagnosed T2D patients, as evidenced by reductions in FBG and HbA1c levels. Additionally, both drugs significantly reduced the HSI, body weight, and BMI, with metformin showing a more pronounced effect on weight and BMI. Both vildagliptin and metformin effectively decreased hepatic steatosis in T2D patients. Total cholesterol and LDL are important predictors of hepatic steatosis.

**Trial registration:**

Trial Registration ID: UMIN000055121, registered on 30/07/2024 (retrospectively registered).

## Introduction

Driven by insulin resistance, whether lean or obese, steatohepatitis develops in at least half of all people with type 2 diabetes [1] and is a significant risk factor for future cirrhosis [2], even for individuals without obesity.

The more severe the insulin resistance like in the case of T2D and increase BMI, the more chance of developing HS [3]. The substantial rise in hepatocellular carcinoma (HCC) rates seen in diabetics is thought to be related to steatohepatitis [4].

The clinical practice recommendations of the American Diabetes Association propose the use of glucagon-like peptide-1 (GLP-1) receptor agonists and pioglitazone, two drugs currently available to treat obesity and type 2 diabetes, for the dual purpose of treating non-alcoholic fatty liver disease (NAFLD) in patients with obesity or type 2 diabetes, as endorsed by other medical societies [5–8]. Nevertheless, practitioners have not yet embraced these rules widely. In addition, contrary to current guidelines, statins are often not prescribed when NAFLD is present or are even discontinued if there are mildly elevated plasma aminotransferase levels, even though these people have a high cardiovascular risk [5–8]. Poor comorbidity management in NAFLD patients is thought to be responsible for more than 50% of all-cause deaths [9].

**Fig. 1:**
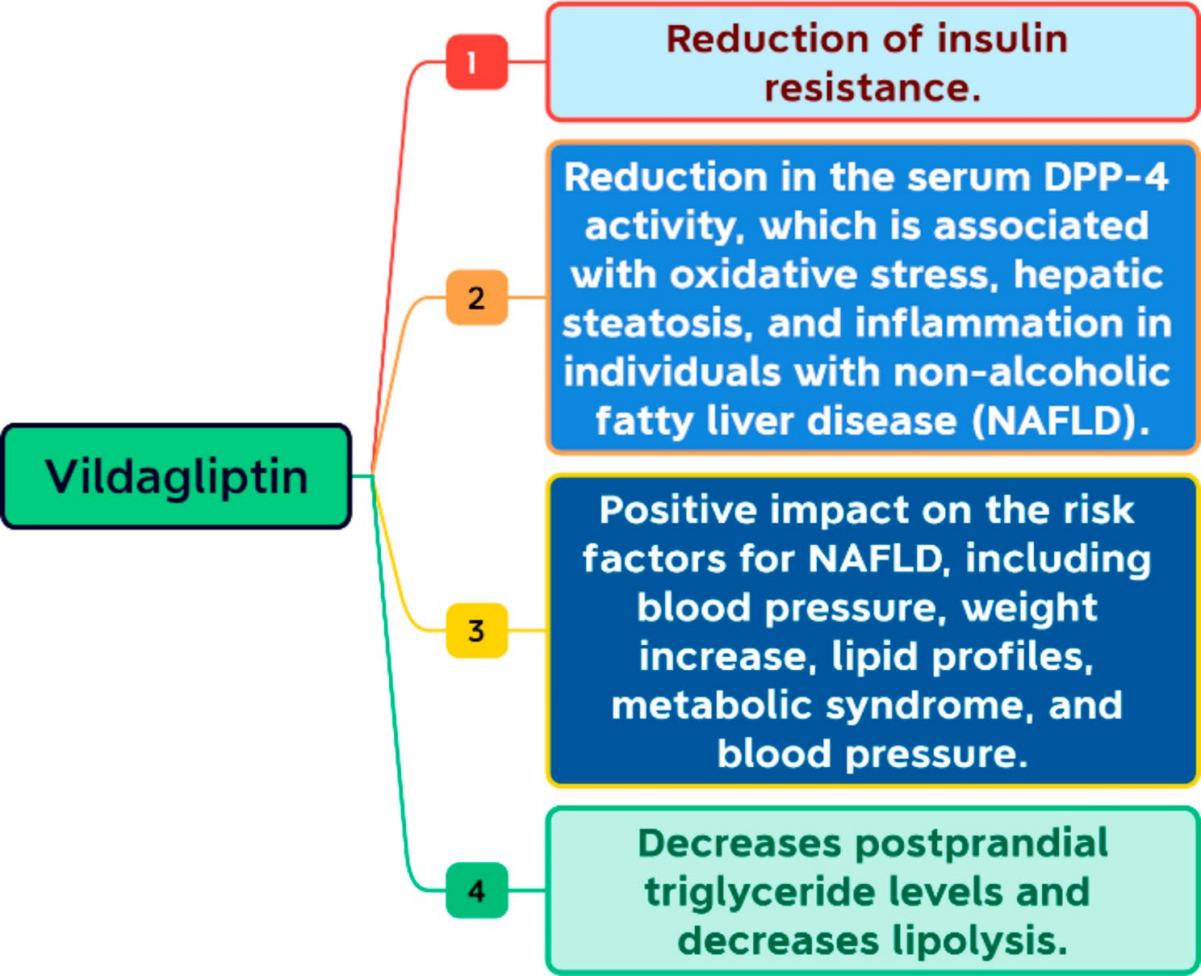
Mechanisms of action of vildagliptin on liver fat

Dipeptidyl peptidase IV (DPP-4) inhibitors were investigated to assess their potential effects beyond glucose regulation. One area of interest was the management of fatty liver disease. The trials investigating certain DPP-4 inhibitors found encouraging results regarding the improvement of liver enzymes and histological responses, despite some contradictory data [10]. Vildagliptin may be useful in treating NAFLD in addition to being a common medication for T2D [11]. Some mechanisms of action of vildagliptin on liver fat are presented in Fig. 1.

One of the most widely used medications for type 2 diabetes (T2D) is metformin, a biguanide derivative that has been prescribed for almost a century [12]. It decreases gluconeogenesis in the liver. This is considered its primary action [13].

**Fig. 2:**
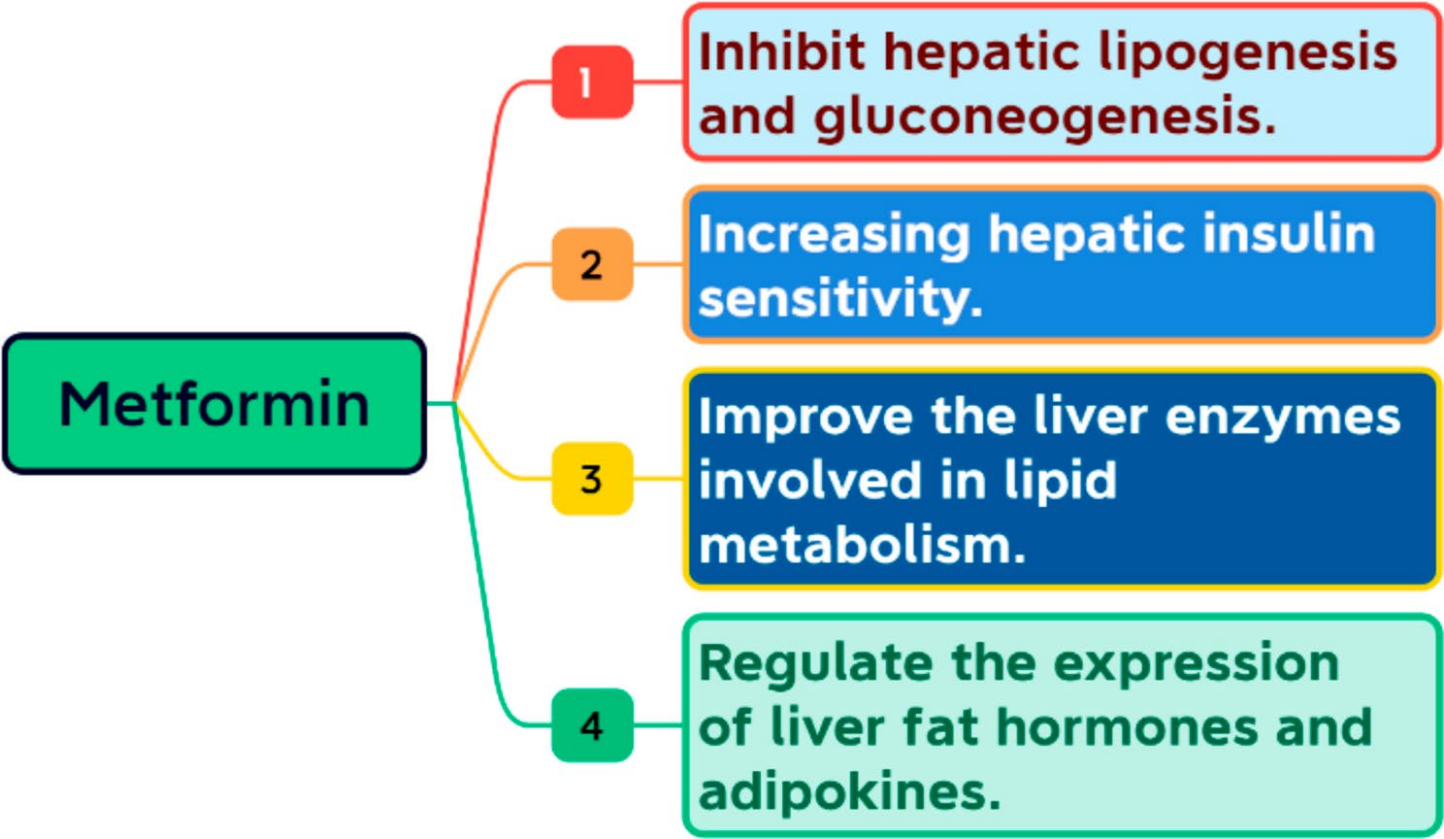
Mechanisms of action of metformin on liver fat

Furthermore, it increases peripheral glucose uptake and utilization, which enhances insulin sensitivity. It may also decrease intestinal glucose absorption [14]. In addition, metformin improves serum levels of alanine transaminase (ALT) and aspartate transaminase (AST) [15]. Some mechanisms of action of metformin on liver fat are presented in Fig. 2.

The main objective of this study was to examine the effect of vildagliptin compared to metformin on liver fat status after a six-month observation period. The null hypothesis of this study is that neither drug has a significant effect on liver fat status, and there is no difference between the two drugs in their effects on liver fat status.

## Methods

### Study design

This study is a 6-months randomized clinical trial adopting an open-label, parallel-group design. Follow-up was performed by both physicians and clinical pharmacists. The aim of the study is to evaluate the effects of vildagliptin and metformin treatments on hepatic steatosis. The study was approved by the ethics committee of Port Said University, and all patients provided written consent to participate.

### Study site

The study was conducted in internal medicine clinics at Minia University Hospital and multiple private clinics concerned with diabetes management in Minia Governorate.

### Participants

**Fig. 3:**
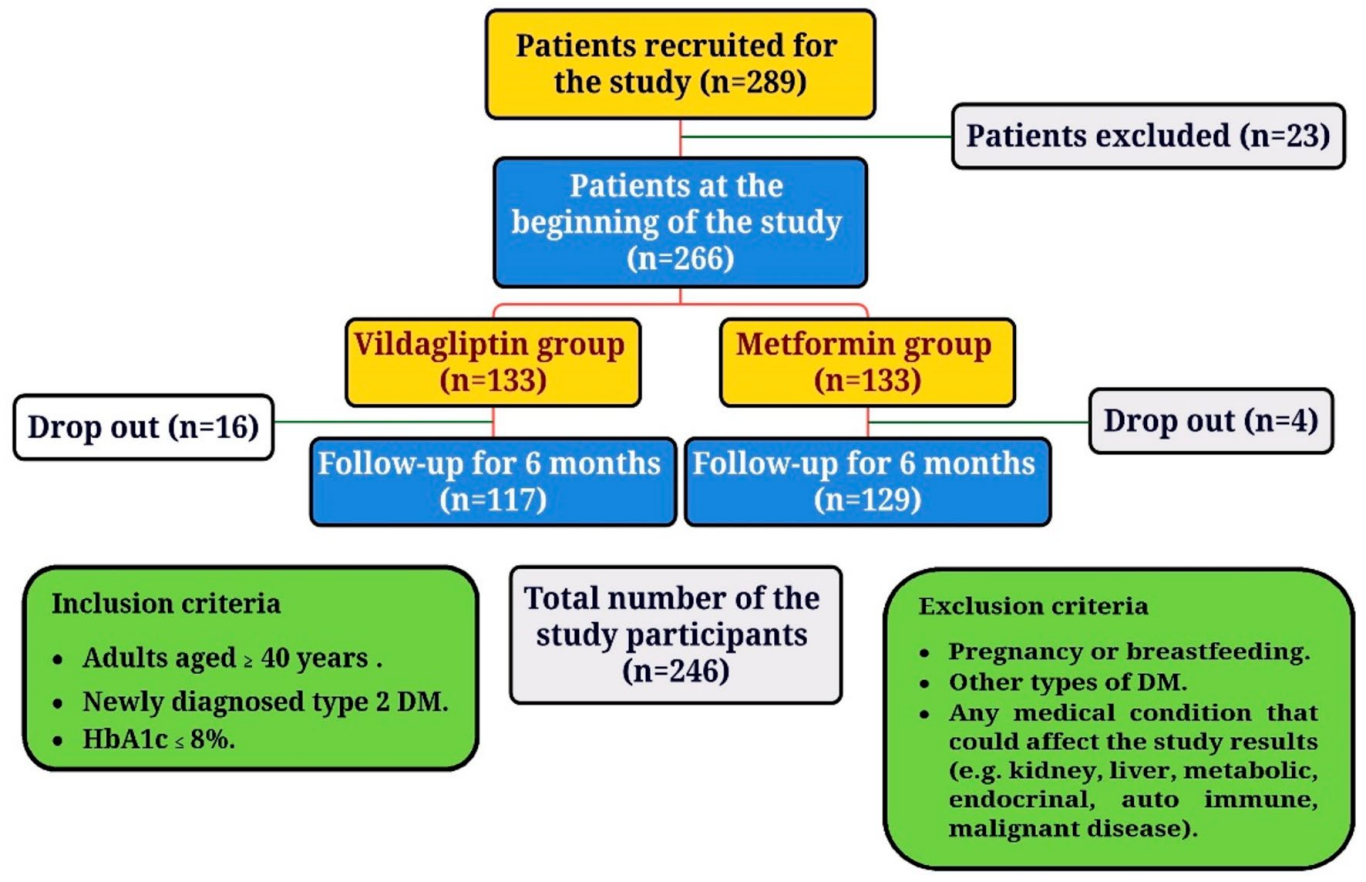
The flowchart of the study

In this study, 289 newly diagnosed type 2 diabetic patients, according to the American Diabetes Association criteria for the diagnosis and classification of diabetes [16], were recruited. After applying the inclusion and exclusion criteria, 23 patients were excluded, leaving 266 patients: 133 in the vildagliptin group and 133 in the metformin group. During follow-up, 16 patients from the vildagliptin group and 4 from the metformin group dropped out. Finally, 246 patients completed the study. The inclusion criteria were adults aged ≥ 40 years, a new diagnosis of type 2 DM, and HbA1c ≤ 8%. The exclusion criteria were pregnancy or breastfeeding, other types of DM, and any medical condition that could affect the study results (e.g., kidney, liver, metabolic, endocrine, autoimmune, or malignant diseases). The main study flowchart is shown in Fig. 3.

### Procedure

Simple randomization was used after patients were matched for age and gender, and they were divided into two groups. We employed a computer-generated randomization sequence to ensure each patient had an equal chance of being allocated to either group. In the first group, patients were prescribed vildagliptin orally at 50 mg twice daily. In the second group, patients were prescribed metformin orally at an initial dose of 500 mg twice daily with meals. The dosage could be gradually increased by 500 mg per week, as tolerated, based on glycemic response and individual tolerance, with a maximum daily dose of 2000 mg.

Patients underwent clinical assessment every two weeks by a specialized internal medicine or endocrinologist physician and a clinical pharmacist for six months.

### Liver ultrasound

**Fig. 4:**
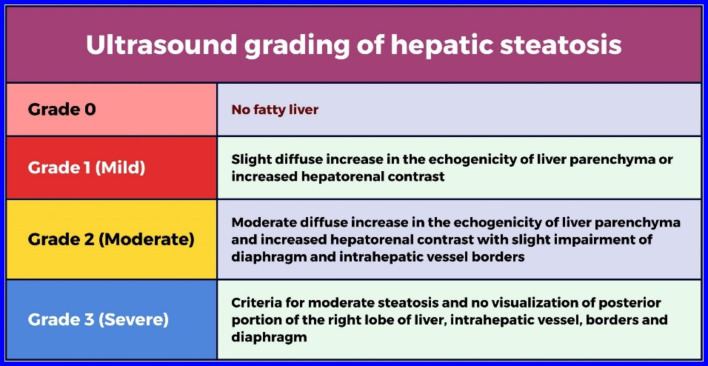
Ultrasound grading of hepatic steatosis

Liver ultrasound was performed at baseline and six months post-treatment and evaluated by a specialized radiologist. NAFLD classification was based on the severity of hepatic steatosis observed on the abdominal ultrasound according to the criteria presented in Fig. 4 [11].

### Outcome measurements

The primary outcomes are glycemic control, measured by fasting blood glucose (FBG) levels, glycated hemoglobin (HbA1c) levels, and the hepatic steatosis index (HSI). The HSI is a convenient tool based on body mass index (BMI), alanine transaminase (ALT), and aspartate transaminase (AST) levels. BMI is calculated as weight (kg) divided by the square of height (m). Hepatic steatosis index (HSI) values of 36 and above indicate that a positive diagnosis of hepatic steatosis is highly likely. HIS = 8 × ALT/AST ratio + BMI (+ 2, if DM; +2, if female) [17].

Secondary outcomes included anthropometric measurements such as weight loss, BMI, waist circumference (WC), and hip circumference (HC). Waist circumference was measured with a non-stretchable tape measure placed horizontally around the body at a point midway between the lowest rib border and the iliac crest. Hip circumference was measured with a non-stretchable tape measure around the widest part of the hips while the participant stood upright. Additionally, the lipid profile was measured for all participants.

### Data analysis

Data were analyzed using SPSS v26 software. Continuous variables were tested for normal distribution using the Kolmogorov-Smirnov test. Descriptive statistics were used to summarize the data. The chi-square test was used for comparing qualitative data between two groups. An independent samples t-test was used to compare quantitative data between the two groups. A paired t-test was used for comparing paired quantitative data in the study. The Wilcoxon signed-rank test was used for comparing paired ordinal data. A *p*-value of < 0.05 was set as the threshold for statistical significance. MedCalc v20.3 software was used to generate a receiver operating characteristic (ROC) curve.

## Results

In this study, we investigated the effects of vildagliptin and metformin on the hepatic steatosis index (HSI) and the grading of hepatic steatosis (HS) by ultrasound in 246 newly diagnosed type 2 diabetes (T2D) patients. The mean age was 48.25 ± 3.95 years. Females represented 67.89% of the sample.

Table 1 shows the demographic data of the studied sample. Health care workers comprised 10.16% of the study sample. Married participants made up 82.11%. Participants from urban areas constituted 69.92% of the sample. Non-educated participants accounted for 7.32%. Participants with no special habits represented 51.63%. A positive family history of diabetes mellitus (DM) was found in 32.93% of participants, while a positive family history of hypertension was present in 15.04% of cases.

**Table 1:** Demographic characteristics of the study sample

Sociodemographic characteristics	n	%
**Age/year**		
*Mean ± SD*	48.25 ± 3.95	
**Gender M/F**		
*Male/Female*	79/167	32.11/67.89
**Occupation**		
Health care workers / Others	25 / 221	10.16 / 89.84
**Marital status**		
Married / Single, divorced or widow	202 / 44	82.11 / 17.89
**Residence**		
Urban / Rural	172 / 74	69.92 / 30.08
**Educational level**		
None	18	7.32
School	109	44.31
University	119	48.37
**Special habits**		
None	127	51.63
Tea or coffee > 3 times/D	107	43.49
Smoking	12	4.88
**Family history of DM**		
Yes / No	81 / 165	32.93 / 67.07
**Family history of HTN**		
Yes / No	37 / 209	15.04 / 84.96

SD, standard deviation; DM, diabetes mellitus; HTN, hypertension

Table 2 shows the comparison of baseline clinical and laboratory data between the two groups. There is no statistically significant difference between the groups in terms of age, heart rate, systolic blood pressure, diastolic blood pressure, ALT, AST, urea, creatinine, total cholesterol, triglycerides, HDL, and LDL.

**Table 2:** Baseline clinical and laboratory data in the study groups

	Total	Vildagliptin	Metformin	*p*
	(n = 246)	(n = 117)	(n = 129)	
	Mean ± SD	Mean ± SD	Mean ± SD	
Age	48.25 ± 3.95	48.42 ± 4.09	48.1 ± 3.8	0.53
HR (beat/ min)	83.17 ± 6.7	82.78 ± 6.95	83.53 ± 6.48	0.38
SBP (mmHg)	131.3 ± 17.3	131.96 ± 17.1	130.73 ± 17.5	0.58
DBP (mmHg)	74.9 ± 9.4	75.5 ± 9.8	74.5 ± 8.9	0.4
ALT (u/l)	25.14 ± 11.9	24.7 ± 11.4	25.53 ± 12.4	0.58
AST (u/l)	27.65 ± 12.1	27.23 ± 12.1	28 ± 12.2	0.6
Urea (mg/dl)	16.67 ± 5.8	17.05 ± 5.8	16.32 ± 5.7	0.32
Creatinine (mg/dl)	0.929 ± 0.17	0.95 ± 0.16	0.91 ± 0.18	0.08
Total cholesterol (mg/dl)	167.28 ± 37.1	166.8 ± 36.9	167.7 ± 37.4	0.84
Triglycerides (mg/dl)	140.22 ± 63.5	143.9 ± 65.8	136.9 ± 61.5	0.39
HDL- cholesterol (mg/dl)	40.96 ± 5.78	41 ± 6.1	40.92 ± 5.48	0.91
LDL-cholesterol (mg/dl)	98.28 ± 38.8	97 ± 39.29	99.4 ± 38.54	0.63

The *p*-value was considered significant if it was less than 0.05. HR, heart rate; SBP, systolic blood pressure; DBP, diastolic blood pressure; mmHg, millimeters of mercury; HR, heart rate; bpm, beats per minute; HDL, High-density lipoprotein; LDL, Low-density lipoprotein

Table 3 shows that in the vildagliptin group, the baseline mean ± standard deviation (M ± SD) values for FBG, HbA1c, weight, BMI, WC, HC, and HSI levels were 192.55 ± 69.2, 7.31 ± 0.47, 68 ± 13.7, 24.57 ± 4.56, 99.04 ± 12.9, 107.7 ± 15.3, and 37.85 ± 10.2, respectively. The 6-month follow-up M ± SD values for these levels were 83.75 ± 17.5, 5.55 ± 0.55, 64.23 ± 13.65, 23.21 ± 4.56, 97.97 ± 12.88, 106.04 ± 15, and 36.48 ± 11.94, respectively. All of these variables showed a significant difference between the baseline and 6-month follow-up values (*p* < 0.001).

**Table 3:** Comparison between the outcomes pre and after intervention in the two groups

	Total	Vildagliptin	Metformin	P1
	(n = 246)	(n = 117)	(n = 129)	
	Mean ± SD	Mean ± SD	Mean ± SD	
**FBG (mg/dL)**				
Baseline	193.5 ± 69.4	192.55 ± 69.2	194.36 ± 69.8	0.84
After six months	82.88 ± 17.6	83.75 ± 17.5	82.09 ± 17.8	0.46
p	< 0.001	< 0.001	< 0.001	
**HbA1c (%)**				
Baseline	7.27 ± 0.46	7.31 ± 0.47	7.23 ± 0.46	0.2
After six months	5.53 ± 0.62	5.55 ± 0.55	5.51 ± 0.68	0.55
p	< 0.001	< 0.001	< 0.001	
**Height (cm)**				
Baseline	1.66 ± 0.1	1.66 ± 0.09	1.65 ± 0.08	0.16
**Weight (kg)**				
Baseline	67.16 ± 13.2	68 ± 13.7	66.4 ± 12.79	0.35
After six months	61.37 ± 13.5	64.23 ± 13.65	58.78 ± 12.9	0.001
p	< 0.001	< 0.001	< 0.001	
**Body mass index (kg/m** ^**2**^ **)**				
Baseline	24.54 ± 4.58	24.57 ± 4.56	24.51 ± 4.61	0.9
After six months	22.4 ± 4.6	23.21 ± 4.56	21.67 ± 4.57	0.009
p	< 0.001	< 0.001	< 0.001	
**Waist circumference (cm)**				
Baseline	99 ± 12.75	99.04 ± 12.9	98.98 ± 12.68	0.96
After six months	97.98 ± 12.73	97.97 ± 12.88	97.99 ± 12.66	0.9
p	< 0.001	< 0.001	< 0.001	
**Hip circumference (cm)**				
Baseline	108.14 ± 14.8	107.7 ± 15.3	108.54 ± 14.37	0.66
After six months	106.6 ± 14.78	106.04 ± 15	107.13 ± 14.31	0.56
p	< 0.001	< 0.001	< 0.001	
**Hepatic steatosis index**				
Baseline	38 ± 10.36	37.85 ± 10.2	38.15 ± 10.53	0.82
After six months	36.4 ± 12.14	36.48 ± 11.94	36.34 ± 12.4	0.9
p	< 0.001	< 0.001	< 0.001	

p = comparison of baseline and 6-month follow-up results within the same group; p1 = comparison of baseline and 6-month follow-up results between the vildagliptin group and the metformin group. FBG, fasting blood glucose; HbA1c, glycated hemoglobin; BMI, body mass index; kg/m² = kilograms per square meter

In the metformin group, the baseline M ± SD values for FBG, HbA1c, weight, BMI, WC, HC, and HSI levels were 194.36 ± 69.8, 7.23 ± 0.46, 66.4 ± 12.79, 24.51 ± 4.61, 98.98 ± 12.68, 108.54 ± 14.37, and 38.15 ± 10.53, respectively. The 6-month follow-up M ± SD values for these levels were 82.09 ± 17.8, 5.51 ± 0.68, 58.78 ± 12.9, 21.67 ± 4.57, 97.99 ± 12.66, 107.13 ± 14.31, and 36.34 ± 12.4, respectively. All of these variables showed a significant difference between the baseline and 6-month follow-up values (*p* < 0.001).

The 6-month follow-up weight and BMI levels in the metformin group were significantly lower than those in the vildagliptin group (*p* = 0.001 and *p* = 0.009, respectively).

Table 4 shows that there is no significant difference between the two groups in terms of the frequencies of fatty liver grading on ultrasound at baseline or after six months of follow-up (*p* = 0.86 and 0.8, respectively). At baseline, the frequencies of grades 0, 1, 2, and 3 in the vildagliptin group were 62.39%, 19.66%, 11.97%, and 5.98%, respectively, while in the metformin group, they were 59.69%, 23.25%, 10.08%, and 6.98%, respectively. After six months of follow-up, the frequencies of grades 0, 1, 2, and 3 in the vildagliptin group were 70.94%, 17.09%, 8.55%, and 3.42%, respectively, while in the metformin group, they were 70.54%, 18.61%, 9.3%, and 1.55%, respectively. This table indicates that both vildagliptin and metformin led to a significant decrease in hepatic steatosis grading on ultrasound.

**Table 4:** The effect of vildagliptin versus metformin on hepatic steatosis grading by ultrasound

	Grade	Total		Vildagliptin		Metformin		P1
		(n = 246)		(n = 117)		(n = 129)		
		n	%	n	%	n	%	
**Baseline**								
	0	150	60.98	73	62.39	77	59.69	
	1	53	21.54	23	19.66	30	23.25	0.86
	2	27	10.98	14	11.97	13	10.08	
	3	16	6.5	7	5.98	9	6.98	
**After six months**								
	0	174	70.73	83	70.94	91	70.54	
	1	44	17.89	20	17.09	24	18.61	0.8
	2	22	8.94	10	8.55	12	9.3	
	3	6	2.44	4	3.42	2	1.55	
**p**		< 0.001		< 0.001		< 0.001		

p = comparison of baseline and 6-month follow-up results within the same group; p1 = comparison of baseline and 6-month follow-up results between the vildagliptin group and the metformin group

Table 5 shows a significant correlation between the hepatic steatosis index (HSI) and HbA1c, BMI, WC, and HC (*p* < 0.001 for all), as well as FBG (*p* = 0.008). However, no significant correlation was found between the hepatic steatosis index and age (*p* = 0.90).

**Table 5:** Correlation between the hepatic steatosis index and laboratory data

Variables	Hepatic steatosis index	
	r	P
Age (years)	0.007	0.9
FBG (mg/dl)	0.169	0.008
HbA1c (%)	0.28	< 0.001
BMI (kg/m^2^)	0.467	< 0.001
Waist circumference (cm)	0.472	< 0.001
Hip circumference (cm)	0.364	< 0.001

r, Pearson’s correlation coefficient; FBG, fasting blood glucose; HbA1c, glycated hemoglobin; BMI, Body mass index

**Fig. 5:**
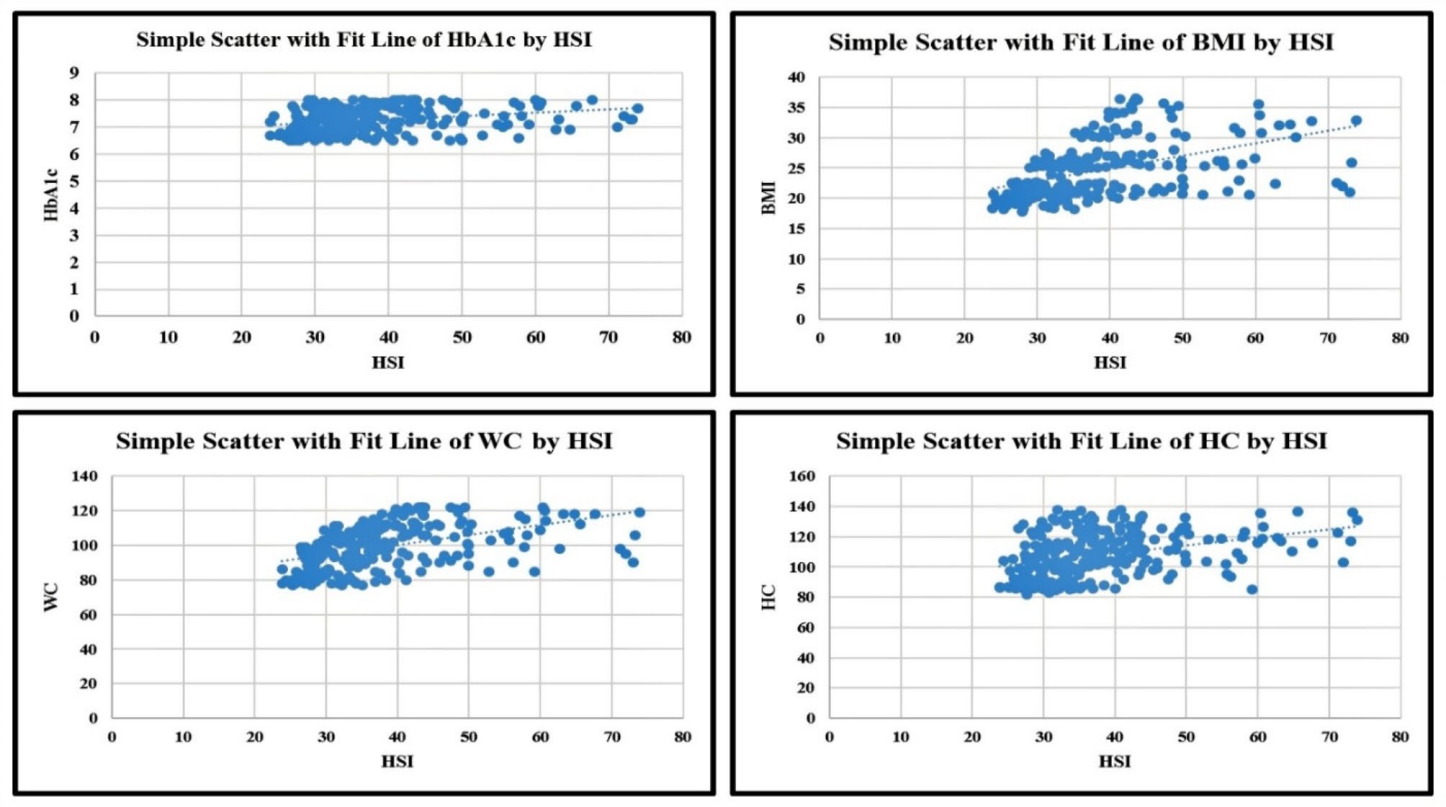
Correlation between the hepatic steatosis index and clinical and laboratory data. *Abbreviations* HIS, hepatic steatosis index; HbA1c, glycated hemoglobin; BMI, Body mass index; WC, Waist circumference; HC, Hip circumference

A significant correlation between HbA1c, body mass index, waist circumference, hip circumference, and the hepatic steatosis index is demonstrated in Fig. 5.

Table 6 shows the relationship between hepatic steatosis index levels and the demographic data of the studied sample. There is no statistically significant association between the demographic data and hepatic steatosis index levels.

**Table 6:** Relationship between hepatic steatosis index levels and the demographic data of the studied sample

	N	Mean ± SD	*p*
**Gender**			
Male	79	37.23 ± 10.7	0.42
Female	167	38.38 ± 10.2	
**Occupation**			
Health care workers	25	38.12 ± 10.7	0.95
Others	221	38 ± 10.4	
**Marital status**			
Married	202	37.85 ± 10.1	0.61
Single, divorced or widow	44	38.74 ± 11.5	
**Residence**			
Urban	172	37.76 ± 9.9	0.57
Rural	74	38.59 ± 11.4	
**Special habits**			
None	127	37.37 ± 9.58	0.32
Tea or coffee > 3 times – Smoking	119	38.69 ± 11.13	
**Family history of diabetes mellitus**			
No	165	38.34 ± 10.17	0.47
Yes	81	37.32 ± 10.77	
**Family history of hypertension**			
No	209	38.32 ± 10.54	0.26
Yes	37	36.24 ± 9.19	

Table 7 presents the relationship between hepatic steatosis, as assessed by the hepatic steatosis index (HIS = 36 and above), and lipid profile variables in the studied sample. Each lipid variable (Total cholesterol, triglycerides, high-density lipoprotein [HDL], and low-density lipoprotein [LDL]) has an associated optimal cutoff point for predicting hepatic steatosis. Total cholesterol levels above 165 mg/dL demonstrate strong predictive value with an AUC of 0.83, indicating high accuracy, and achieve a sensitivity of 77.1% and specificity of 83.6% (*p* < 0.001). Triglycerides with a cutoff above 123 mg/dL show lower predictive performance, with an AUC of 0.59, sensitivity of 61.02%, and specificity of 61.72% (*p* = 0.013). For HDL, a cutoff of 36 mg/dL or lower correlates with hepatic steatosis with an AUC of 0.71, though with lower sensitivity (39.8%) but very high specificity (98.4%), indicating it may be a strong indicator of non-steatosis when HDL is above 36 mg/dL (*p* < 0.001). LDL levels above 108 mg/dL also predict hepatic steatosis with a high AUC of 0.8, sensitivity of 61%, and specificity of 90.6% (*p* < 0.001). Overall, Total cholesterol and LDL appear to be more accurate predictors of hepatic steatosis in this sample, as indicated by their high AUC, sensitivity, and specificity values.

**Table 7:** Relationship between hepatic steatosis according to HSI levels, and lipid profile of the studied sample

Variable	Cut-off point mg/dl	AUC	*p*	Sensitivity %	Specificity %
Total cholesterol	> 165	0.83	< 0.001	77.1	83.6
Triglycerides	> 123	0.59	0.013	61.02	61.72
High density lipoprotein	≤ 36	0.71	< 0.001	39.8	98.4
Low density lipoprotein	> 108	0.8	< 0.001	61	90.6

**Fig. 6:**
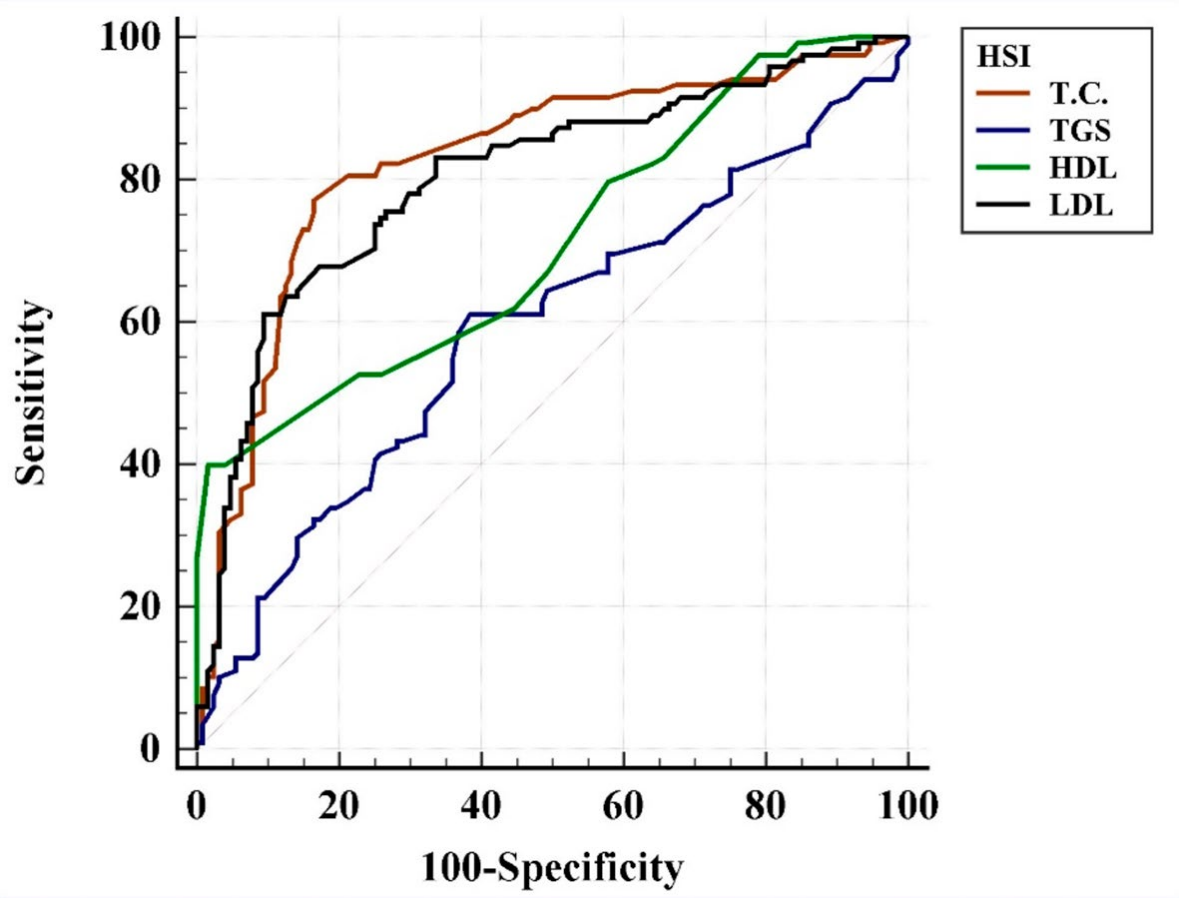
ROC curve analysis of the relationship between hepatic steatosis according to HSI levels and the lipid profile of the study participants. *Abbreviations* HIS, hepatic steatosis index; T.C., Total cholesterol; TGS, Triglycerides; HDL, High-density lipoprotein; LDL, Low-density lipoprotein

A strong ability to discriminate between positive and negative NAFLD patients based on the hepatic steatosis index (HSI) across all four-lipid profile variables is demonstrated in Fig. 6. The area under the curve (AUC) values for total cholesterol (TC), triglycerides (TG), high-density lipoprotein (HDL), and low-density lipoprotein (LDL) were 0.83, 0.59, 0.71, and 0.80, respectively, indicating good overall model performance. The curve lies significantly above the random chance line, confirming the model’s effectiveness in distinguishing between positive and negative cases for these lipid profile variables.

## Discussion

In the current study, 246 newly diagnosed T2D patients were enrolled and divided into two groups. Through randomization, 117 participants were allocated to the vildagliptin therapy group, while the remaining 129 participants were assigned to the metformin therapy group. We investigated the effects of vildagliptin and metformin on the HSI and the grading of hepatic steatosis by ultrasound. Both drugs showed a significant decrease from baseline to the 6-month follow-up in FBG, HbA1c, weight, BMI, WC, HC, and HSI. At the 6-month follow-up, weight and BMI levels in the metformin group were significantly lower than those in the vildagliptin group, indicating a potentially greater effect of metformin on these variables. Additionally, both vildagliptin and metformin led to a significant decrease in hepatic steatosis grading on ultrasound, with no significant difference between the two drugs. This study revealed a significant correlation between the HSI and HbA1c, BMI, WC, HC, and FBG. However, no significant correlation was found between HSI and age. In this study, total cholesterol and LDL appeared to be more accurate predictors of hepatic steatosis, as indicated by their high AUC, sensitivity, and specificity values.

Nonalcoholic fatty liver disease (NAFLD) is quite common and closely linked to type II diabetes, metabolic syndrome, and insulin resistance. A major contributing factor to the complex etiopathogenesis of this illness is insulin resistance, which leads to the accumulation of free fatty acids in liver cells, causing lipotoxicity, inflammation, and fibrosis [18]. A meta-analysis of nearly 50,000 patients from 80 studies revealed that the global prevalence of NAFLD in T2D was as high as 55.5%. Additional studies showed that up to 59.67% of T2D patients and even 77.87% of obese T2D patients had NAFLD [19].

The present study revealed that the mean age of the newly diagnosed type 2 diabetic patients was 48.25 years. In a separate cross-sectional analysis of 8695 new type 2 diabetic patients, the mean age at T2DM diagnosis was 45.1 years in men and 45.0 years in women [20]. Additionally, a study sample including 3,022 participants (1586 men and 1436 women) found that the overall mean age at diabetes diagnosis was 49.9 years (95% CI, 49.2–50.7 years) [21].

Health care workers constituted 10.16% of the current study sample. Many studies have revealed an association between the prevalence of type 2 diabetes mellitus (DM) and specific occupations. According to one study, doctors are less likely to develop diabetes than the general population. However, surgeons and emergency room doctors are more likely to develop the disease compared to other specialist physicians [22]. These variations in DM prevalence are likely due to significant differences in lifestyle risk factors. Workplace interventions that encourage physical activity and assist workers in these occupations with weight loss could result in significant improvements in health [23]. In a cross-sectional study of 299 participants, sociodemographic factors and risk variables for type 2 diabetes were estimated. According to the findings, women made up 63.9% of the sample, while nursing staff comprised 40.5%. The study found that nursing personnel have a higher chance of developing diabetes compared to other health professionals [24].

The percentage of married participants was 82.11% in the present study. Another study that examined the incidence of T2DM found that widowed women had a decreased chance of developing T2DM compared to married women [25]. Additionally, another study found that, despite significant weight gain, individuals who remained married had a much lower chance of developing diabetes than those who got divorced [26].

In the current study, participants from urban areas comprised 69.92%. A meta-analysis demonstrated a significant correlation between urban living and a 40% higher incidence of type 2 diabetes, especially in low- and middle-income countries [27].

Non-educated participants made up 7.32% of the present study. Diabetes prevalence was high in 29 low- and middle-income countries and increased as World Bank income group levels rose. Regardless of BMI, the risk of diabetes was highest among those with higher educational attainment [28].

Participants with no special habits constituted 51.63% of the current study. A study revealed that, in middle-aged men with normal weight, cigarette smoking has a negative association with the prevalence of type 2 diabetes but is positively correlated with an increased risk of stroke [29].

A positive family history of DM was found in 32.93% of participants in the present study. There is a 41.1% chance of diabetes among participants with diabetic fathers and a 39.3% chance among participants with diabetic mothers [30]. Another study found that the chance of developing type 2 diabetes increased by 2.5 times when there was even one family member with the illness [31].

In the present study, there was a significant decrease in glycemic control elements (FBG and HbA1c) after the follow-up period for both the vildagliptin and metformin groups. Vildagliptin increases the reactivity of both α- and β-cells to glucose, enhancing islet function in type 2 diabetic patients [32]. It prolongs the half-life of glucagon-like peptide, which results in the suppression of glucagon secretion and stimulation of insulin release [33]. It also has a low risk of causing hypoglycemia. Vildagliptin was found to be beneficial in lowering HbA1c levels in individuals who had not previously taken medication, with a 0.4% reduction from baseline over the course of a 4-week study. Patients on DPP-4 inhibitors treatment also showed lower glucagon levels during meals, a finding not previously observed in animals [34]. Furthermore, vildagliptin (50 mg twice daily) was found to be as effective as pioglitazone (30 mg once daily) in decreasing HbA1c levels, and a higher proportion of vildagliptin-treated patients compared to those receiving glimepiride were able to achieve their target HbA1c without experiencing hypoglycemia [35].

At the time of T2D diagnosis, patients are often started on metformin monotherapy along with lifestyle modifications, unless they have poor glycemic control, contraindications, or metformin intolerance. Many guidelines recommend using SGLT-2 inhibitors and GLP-1 receptor agonists as first-line monotherapy in patients with atherosclerotic cardiovascular disease or at high cardiovascular risk [36]. A meta-analysis of 134 trials assessing monotherapy treatment for diabetes in patients who had not previously taken antidiabetic medication observed an effective reduction in HbA1c levels of up to 1.5% with metformin treatment [37].

The present study revealed that vildagliptin and metformin effectively decreased anthropometric measurements such as weight, BMI, WC, and HC. Additionally, metformin was more effective than vildagliptin in reducing both weight and BMI. A randomized, placebo-controlled trial showed that body weight significantly decreased during twelve weeks of vildagliptin treatment compared to placebo (*p* = 0.04) and that BMI was also significantly lower with vildagliptin than with placebo (*p* = 0.028) [11]. In a study examining the effect of vildagliptin on fatty liver, the mean body weight decreased by 1.6 ± 0.5 kg from baseline (*P* = 0.002) in the vildagliptin group, compared to a decrease of 0.4 ± 0.5 kg from baseline (*P* = 0.41) in the placebo group over a 6-month period [38]. A meta-analysis revealed that metformin treatment resulted in about a one-unit reduction in BMI at the end of the treatment period, with the decrease being most significant in patients with simple obesity [39].

The current study showed that both vildagliptin and metformin reduce liver fatty infiltration, as evidenced by decreased fatty liver grading on ultrasound and a lower hepatic steatosis index (HSI). A randomized, placebo-controlled trial investigating the effect of vildagliptin in patients with NAFLD found significant regression in fatty liver grading after 12 weeks of treatment with vildagliptin compared to placebo [11]. Another study demonstrated that vildagliptin treatment resulted in a significant decrease in hepatic triglyceride levels over 6 months, independent of changes in body weight. There was no alteration in peripheral insulin sensitivity [40]. Vildagliptin exerts its beneficial effects in patients with NAFLD in several ways. It may improve insulin resistance, which is an important metabolic defect in NAFLD patients [41]. Additionally, it lowers blood DPP-4 activity, as hepatic steatosis is correlated with elevated DPP-4 levels in NAFLD patients [11]. Reducing DPP-4 levels helps decrease inflammation in NAFLD patients, which is one of the disease’s characteristic features [42]. Moreover, vildagliptin has beneficial effects on various risk factors associated with NAFLD, as shown in both clinical and animal studies. These include improvements in metabolic syndrome, blood pressure, weight gain, and lipid profiles [43]. Furthermore, vildagliptin possesses anti-inflammatory and antioxidant properties, which are important given that oxidative stress and inflammation are significant factors in the development and progression of NAFLD [42].

Metformin may help manage NAFLD by reducing insulin resistance (IR) and hyperinsulinemia [44]. Many studies have suggested that metformin might delay the onset and progression of NAFLD [45]. Additionally, numerous studies have linked metformin’s anti-inflammatory properties to its ability to alleviate NAFLD [46, 47].

The current study shows a significant correlation between the hepatic steatosis index and HbA1c, BMI, WC, and HC (*p* < 0.001 for all), as well as FBG (*p* = 0.008). Diabetes and obesity have been identified as strong indicators of NAFLD [48]. Consequently, individuals with NAFLD also have higher HbA1c values. Similarly, several body measurement indicators, including BMI and waist hip ratio, have been associated with type 2 diabetes, insulin resistance, and NAFLD [49].

The present study showed that the lipid profile is a good predictor of liver fat state according to HSI. An Iranian cohort study on biochemical markers and lipid profile in NAFLD found that an increase in FBS, TC, LDL/HDL ratio, TC/HDL ratio, AST/ALT ratio, GGT, ALT, and AST (OR > 1), and a reduction in HDL (OR < 1) significantly increased the likelihood of developing NAFLD (*p* < 0.05) [50].

### Limitations

The relatively short duration of the study and the absence of multiple longitudinal data over a longer period are the main limitations of this study. Future research with larger sample sizes and longer follow-up periods is needed to confirm the study results.

## Conclusion

Concomitant fatty infiltration of the liver occurs to varying degrees with type 2 diabetes. Both vildagliptin and metformin can be used not only to control blood glucose levels but also to potentially reduce the degree of liver fatty infiltration. While the exact mechanisms of action of vildagliptin and metformin are not fully understood, a reduction in anthropometric measurements, such as body weight and body mass index, may be an indirect mechanism for this effect. It is important to monitor all lipid profile elements, especially in newly diagnosed type 2 diabetic patients, as they are potential risk factors and reliable predictors for hepatic steatosis in the present study.

## Data Availability

No datasets were generated or analysed during the current study.
